# Modeling the Pharmacotherapy Cost and Outcomes of Primary Open-Angle Glaucoma With Dry Eye

**DOI:** 10.3389/fpubh.2019.00363

**Published:** 2019-12-20

**Authors:** Konstantin Tachkov, Anton Vassilev, Stanislava Kostova

**Affiliations:** ^1^Department of Organization and Economics of Pharmacy, Faculty of Pharmacy, Medical University of Sofia, Sofia, Bulgaria; ^2^Department of Ophthalmology, Medical University of Sofia, Sofia, Bulgaria

**Keywords:** glaucoma, pharmacotherapy, dry eye, cost-effectiveness, decision tree model

## Abstract

We aimed to analyze and model the cost and results of current outpatient pharmacotherapy practice in patients with primary open-angle glaucoma concomitant with dry-eye disease (POAG+DE). The point of view is that of the health care system and patients, and the time horizon was 1 year. Data were collected through a prospective, observational, real-life study of therapy practice in patients admitted to the specialized ophthalmology clinic at the Alexandrovska University Hospital in Sofia. Pharmacotherapy was recorded and analyzed by therapeutic group and INN. The probability of being prescribed preservative-free or non-free formulations was calculated, as were the cost of yearly therapy, reimbursed cost, and patient co-payment. A decision tree exploring the cost-effectiveness of preservative-free and preservative non-free formulations was built. Outcomes were recorded through three tests measuring tear film stability: TMS, NIBUT Ave, and ST. TMS values below 3, ST above 10 mm, and NIBUT Ave above 14 s were considered as indicators of good disease control. A total of 140 eyes were diagnosed with POAG, of which 64 had concomitant dry-eye disease and were included in the analysis. Monotherapy was prescribed to 34: 14 on preservative-free formulations and 20 on non-free. Meanwhile, 30 eyes received combination therapy: six on preservative-free and 24 on non-free. The monotherapy product was most commonly Prostaglandin Analogs (PG−73.5%), followed by beta-blockers (BB−26.5%). No carbonic anhydrase inhibitors (Ca AA) or alpha-2 adrenergic agonists (alfa 2 AA) were prescribed as monotherapy. The majority of patients showed poor disease control according to all three measures. The incremental cost-effectiveness ratio (ICER) was 744 BGN for TMS and 131 BGN for NUBIT for each successfully controlled eye—far below three times GDP per capita. For ST, the ICER was negative, benefiting non-free formulations. Therapy of POAG+DED with preservative-free formulations is cost-effective according to the WHO threshold of three times GDP. The median costs of the two treatment modalities were similar. Current practice shows that patients experience a higher burden in terms of co-payment than do institutions such as the NHIF.

## Introduction

Glaucoma, a multi-factorial illness, has been experiencing increasing incidence rates, which have reached 3.54% globally, making it the second leading cause of blindness. The disease afflicts mostly women (1), with the most affected demographic being people aged 40–60 years (2). Primary open-angle glaucoma (POAG) is a progressive, chronic optic neuropathy, the progression of which can lead to loss of retinal ganglia cells and subsequent loss of the field of vision with a risk of complete loss of vision (3, 4).

Dry-eye syndrome is also a multi-factorial illness of the eye lining. It is caused by impaired homeostatic regulation of the tear-film, with accompanying eye symptoms. The etiological reasons for its development include hyperosmolarity, impairment, and inflammation of the ocular surface and neuro-sensory abnormalities (5). The incidence rate is 2.7% for people up to 34 years and 18.6% for people over 75.

Previous studies suggest that 34.9–47.5% of patients with glaucoma also develop dry eye (6). The disease can be influenced to a large degree by the choice of medicinal treatment, especially by those formulations that contain preservatives. A recent review of the German glaucoma register by Erb et al. established that, among glaucoma patients, 50.9% of those who had been prescribed preservative containing monotherapy developed concomitant dry eye disease. For combination therapy, this percentage increased to 65.3% (7). As age progresses, so does the likelihood of developing dry eye, whereby another major factor is the duration of disease. If the POAG duration has been ≤ 1 y, it can reach 45%, and it can reach 59% if the disease has been present for over a year. A study published in 2007 established an 85% association of dry eye and glaucoma for Bulgarian patients (8).

Treatment with preservative-free formulations lowers the risk of developing dry-eye syndrome in patients with POAG (9, 10), but the cost-effectiveness of therapy has not yet been studied, which prompted our interest in this topic. The most widely used preservatives in glaucoma treatments include Benzalkonium chloride (BAK), stabilized oxychloro complex, and ionic-buffered preservatives. It has previously been established that long-term use of such preservatives can lead to the development of ocular surface disease (11–13). The development of dry-eye syndrome seems to be precipitated by a disruption in the ocular microenvironment such as ocular surface disease (14). On the one hand, the disease influences the success of surgery in high-risk patients (15, 15, 16). On the other, it negatively impacts patients' adherence to therapy, the effectiveness of subsequent modalities of treatment, and Quality of Life (17–20) providing further difficulties in the choice of therapy. The first line of treatment typically consists of Prostaglandin Analogs; however, beta-blockers, carbonic anhydrase inhibitors, and alpha-2 adrenergic agonists can also be initiated as monotherapy. Alternatively, for patients without good disease control, these medicines can be offered as a combination therapy. For the purposes of our study, products without the aforementioned preservatives are considered “preservative free” and vice-versa.

Although the topics of glaucoma and BAK toxicity have been widely studied (21, 22), few researchers have focused on dry-eye disease as a comorbidity or the cost-effectiveness of preservative-free formulations when weighed against the risk of developing OSD or DE with preservative-containing formulations (23, 24). Physicians generally avoid prescribing preservative-free medicines due to their higher cost but offer them as an option after clinical diagnosis of dry eye is established. As of the time of writing, according to the National Council on Pricing and Reimbursement (NCPR), a generic brand of preservative-containing latanoprost costs 23.5 BGN (€12), whereas preservative-free latanoprost costs 50.4 BGN (€26) after tax.

## Aim

Since preservative-free medication reduces the risk of dry eye ^9,10^ and offers better disease control, we hypothesized that therapy may actually be cost-effective despite the higher product cost. To our knowledge, this is the first study of its kind to analyze both indications at the same time in a real-world clinical setting.

The decision question stipulated is: “Is it cost-effective to treat all patients with preservative-free formulations or not?”

## Materials and Methods

### Design of the Study

This is a prospective, observational, real-life study of the therapy of POAG+DED (dry-eye disease) in patients admitted at the specialized ophthalmology clinic at the Alexandrovska University Hospital in Sofia. The period of observation was January 2016–September 2017. Of all of the patients admitted at the clinic, those with POAG were extracted, and all with accompanying dry eye were included.

Inclusion criteria were patients aged 30–85 years; visual acuity >0.1 (with or without correction); already established diagnosis of POAG (newly- or previously diagnosed); fundoscopic evidence for glaucoma eye changes; patient consent for participation. Exclusion criteria were ages below 18 years; visual acuity <0.1; recent surgical interventions on the eye; systemic diseases such as hypertension, rheumatoid arthritis, allergy, atopy, and diabetes; active ocular infection; application of other eyedrops.

All patients underwent a full ophthalmological status examination. Meniscometry (LTMH-lower tear meniscus height, mm), non-invasive tear break-up time (NIBUT, s), redness index (RI), and meibography (expressed as MGL %) tests were performed using an Oculus Keratograph 5M. TMS or total meiboscore is a measure of total meibomian gland loss on the lower and upper eyelids and is presented on a scale from 0 to 6. For each eyelid, four intervals describe the percentage loss as a meiboscore (0 to 3). The sum for the two eyelids gives the total meiboscore. Additionally, a Schirmer test (ST, mm) with anesthetic was used to measure tear volume.

Descriptive statistics were obtained with MedCalc™ software, which was used to evaluate the distributions of patients and medication.

The current study was conducted in accordance with the tenets of the Declaration of Helsinki. The study was approved by the ethical committee at the Alexandrovska University Hospital at the Medical University of Sofia on the basis of signed written consent from the patients.

### Pharmacotherapy, Cost, and Outcome Analysis

The pharmacotherapy of the patients was recorded and analyzed by therapeutic group, the INN of the prescribed medicines, preservative-free formulations or not, and the cost of yearly therapy, reimbursed cost, and patient copayment. The preservative content or its absence was assessed based on the summary of product characteristics for each medication. The prices of medicines were gathered from the register of the National Council on Prices and Reimbursement (25). The yearly cost of pharmacotherapy was calculated by multiplying the prescribed dosage regimen with the corresponding unit price of the prescribed dosage form for each product and then calculated for 1 year. The yearly cost of therapy was calculated for each of the affected eyes. All costs are presented in the national currency (BGN) at the exchange rate of 1 BGN = €0.51.

Three of the tests performed were used as outcome measures of disease control for the purposes of the modeling, namely TMS, NIBUT Ave, and ST. TMS values above 3, ST above 10 mm, and NIBUT Ave above 14 s were considered as indicators of good disease control. All of these parameters characterize the ocular surface status of the patients. NIBUT is related to tear film evaporation and is a physiological measure of its lipid phase. TMS represents the morphological substrate for lipid production of the tear film. ST is related to the aqueous phase of the tear film and measures the volume of the basal aqueous tear secretion. Using these three parameters allows the elucidation of the etiology of dry eye and, therefore, the most adequate treatment.

Proof of the effectiveness of therapy was considered to have been obtained when a patient had achieved “good” disease control in all three indicators—TMS, NIBUT Ave, and ST.

### Decision Tree Model and Cost-Outcome Analysis

**Figure 1:**
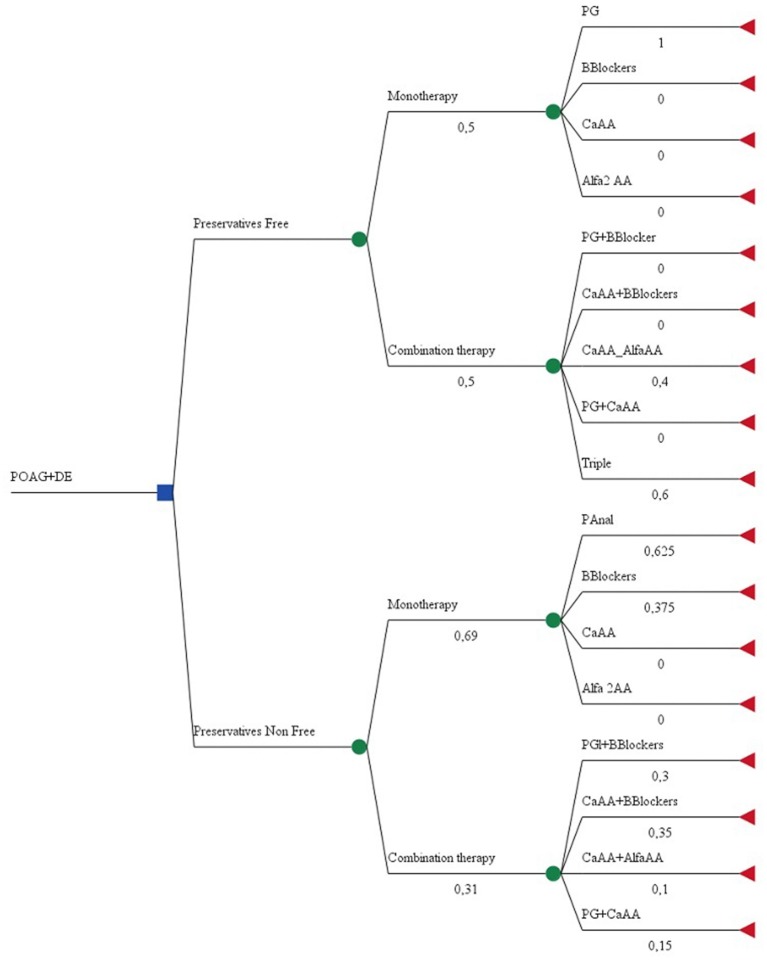
Decision tree model.

A decision tree was built with two branches: preservative-free and preservative non-free formulations. TreeAge Pro™ was used to construct the tree and branches. Initial nodes present the probability of being on mono or combination glaucoma therapy, and the second level depends on the therapeutic class of prescribed medicines and number of INNs in the combination. Probabilities were calculated based on clinical data gathered. The two mutually exclusive criteria were “are preservative-free formulations prescribed?” and “are non-preservative-free formulations prescribed?” The likelihood of an event occurring was calculated via the following formula: $P\left(A\right)=\frac{n\left(A\right)}{n}$. No distinction was made between fixed-dose and non-fixed dose combinations (Figure 1).

### Sensitivity Analysis

The robustness of the results was tested by performing a deterministic one-way sensitivity analysis by varying the cost and effect of prescribing a particular dosage regime within the ±30% interval. The results were displayed through a Tornado Diagram.

## Results

### Patient Demographics

In the period 2016–2018, 251 patients visited the clinic. The total number of eyes analyzed was 502, of which 140 eyes were diagnosed with POAG, and of these, 64 had POAG+DE and met the inclusion criteria. Subsequently, these eyes were divided into two groups: preservative-free and preservative non-free therapy (**Table 2**). The affected eyes were both left (*n* = 30) and right (*n* = 34) and were closely distributed. The gender distribution favored female patients, and all participants were over 50 years of age, pointing toward the significance of the disease for elderly people.

The disease duration for more than half of the patients was over a year, which is sufficient, in most cases, to develop glaucoma and concomitant dry eye as a complication. Tests measuring disease control showed that more patients had poor control than good (Table 1).

**Table 1:** Patient eye characteristics and demographics for POAG+DED.

**Indicator**	**Subgroups**	**Values as a number**	**(%)**
Gender	Males Females	25 39	39.1 60.9
Age	50–60 61–70 71–80 above80	10 28 21 5	15.6 43.8 32.8 7.8
Affected eye	Left Right	30 34	46.9 53.1
Disease duration in years	Less than 1 year Above 1 to 5 Above 5 to10	27 27 10	42.2 42.2 15.6
ST test	Above 10 mm (good control) Below 10 mm (poorcontrol)	15 49	23.4 76.6
TMS	Below 3 (good control) Above 3 (poorcontrol)	23 41	35.9 64.1
NIBUT Ave	Above 14 s (good control) Below 14 s (poorcontrol)	2 62	3.2 96.8

### Pharmacotherapy and Cost Analysis

Mono- or combination therapy for glaucoma was prescribed to a nearly equal number of affected eyes, with a slightly higher share for combinations. Twenty eyes were treated with preservative-free formulations, and ca. 2/3 of them were on monotherapy (*n* = 14). Among the preferred monotherapy alternatives, the distribution was as follows: Prostaglandin Analogs (PG−73.5%), followed by beta-blockers (BB−26.5%). No carbonic anhydrase inhibitors (Ca AA) or alpha-2 adrenergic agonists (alfa 2 AA) were prescribed as monotherapy. However, all of the possible dual combinations and a triple combination were prescribed, including Ca AA and alfa 2 AA (Table 2). Artificial tears were prescribed to all patients as supplemental therapy in order to lessen irritation to the ocular surface, and the costs for the therapy were included in the final analysis.

**Table 2:** Prescribing practice among affected DED eyes.

**Indicator**		**Number in the total group (%)**	**Number of eyes on glaucoma mono-therapy (%)**	**Number of eyes on glaucoma combination therapy (%)**
Total number		64 (100.0)	34 (55)	30 (45)
Number using preservative-free formulations		20 (31.0)	14 (70)	6 (30)
Number using preservative non-free formulations		44 (69.0)	20 (45)	24 (55)
Type of therapeutic group in total population	Beta-blockers Prostaglandins Carbonic AI Alfa2AA		9 25 0 0	
Type of combination	Prostaglandins+Beta-Blockers CaAA+Beta-Blockers CaAA+Prostaglandins CaAA+Alfa 2AA Tripletherapy—CaAA+BB+PG			6 8 5 2 9

The median cost of dry eye therapy with artificial tears was 180 BGN (€90) in addition to the total yearly cost of glaucoma therapy, which was 321 BGN (€161) (Table 3). The total median yearly cost of preservative-free and non-free formulations was almost equal, with only €1 difference between the two. The National Health Insurance Fund currently reimburses only 50% of glaucoma therapy, with patients having to co-pay €80. The dry-eye disease therapy cost is not reimbursed, so the amount of co-pay for patients with concomitant dry-eye disease increases to 339 BGN (€170). Evidently, the financial burden of disease falls heavily on the patients.

**Table 3:** Cost of therapy.

**Type of cost**	**Value, BGN (SD)**
Median, yearly cost of dry-eye therapy	179.93 (9.36)
Total median yearly cost of glaucoma therapy	321.77 (181.00)
Total median yearly cost of therapy with preservative-free formulations (without dry-eye therapy)	320.97 (133.18)
Total median, yearly cost of glaucoma therapy with preservative non free formulations (without dry-eye therapy)	322.14 (203.76)

### Modeling Results

After weighing the cost and effect of therapy for every branch (preservative-free or non-free) with the probability of being on a mono- or combination type therapy and the probability of being prescribed a particular therapeutic class, the estimated costs and results were recorded and are shown in Table 4.

**Table 4:** Incremental cost-effectiveness ratio.

	**Cost**	**Effect**	**Δ Cost**	**Δ**	**ICER**
**PATIENTS WITH GOOD CONTROL ACCORDING TO ST**					
Preservative non-free formulations	444,50	3,52			
Preservative-free formulations	503.80	1.40	59.29	−2.122	−27.94
**PATIENTS WITH GOOD CONTROL ACCORDING TO TMS**					
Preservative non-free formulations	444.50	3.51			
Preservative-free formulations	503.80	3.62	59.29	0.092	644.49
**PATIENTS WITH GOOD CONTROL ACCORDING TO NIBUT**					
Preservative non-free formulations	444.50	0.48			
Preservative-free formulations	503.80	1.00	59.29	0.52	93.48

The incremental cost-effectiveness ratio using ST as a measure of effects was negative, indicating that preservative-free formulations dominate non-free formulations and have lower costs but higher effectiveness in controlling dry-eye disease.

For the other two measures of effect (TMS and NUBIT), the ICER is 744 BGN and 131 BGN for every successfully controlled eye, which is far below three times GDP per capita [the acceptable threshold based on WHO recommendations (26)] (Table 4).

**Figure 2:**
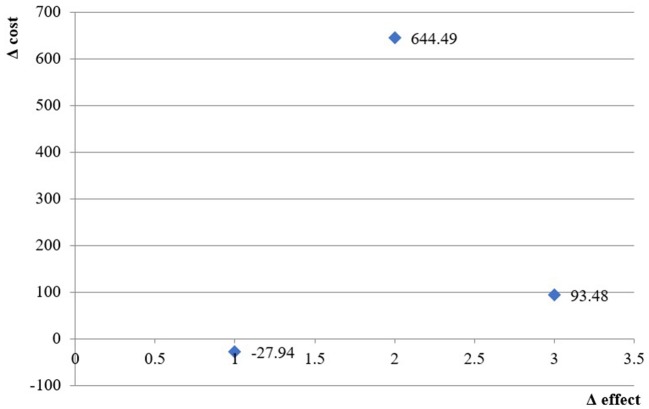
Cost-effectiveness plane.

The cost-effectiveness plane presents the ICER values graphically (Figure 2).

### Sensitivity Analysis

**Figure 3:**
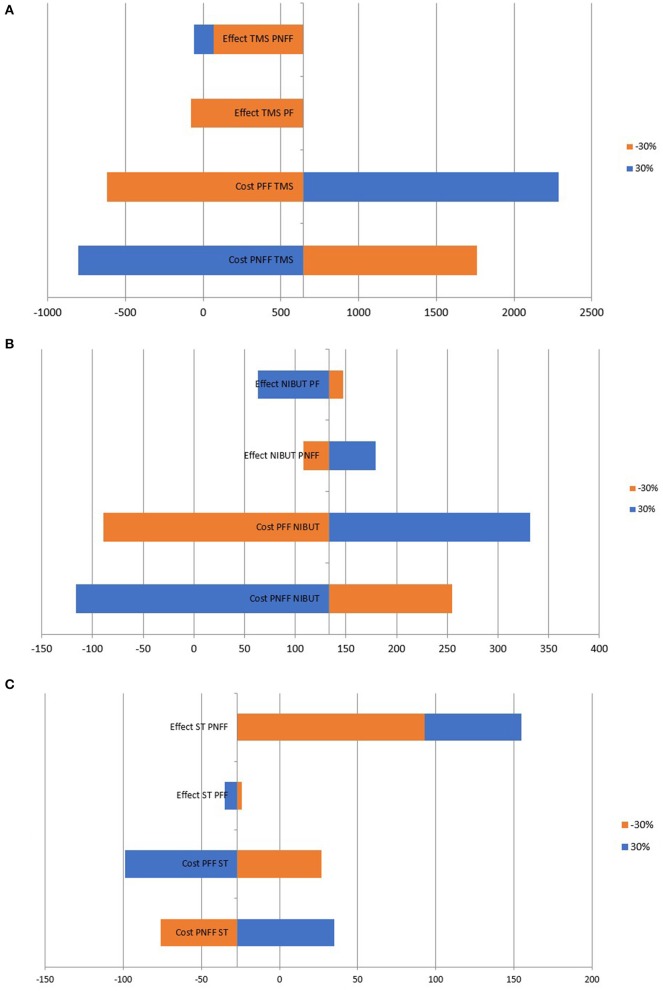
**(A)** TMS. **(B)** NIBUT. **(C)** ST.

Varying the cost and effect within ±30% shows that the most significant factor influencing the ICER are the costs, particularly for the indicators NIBUT and TMS, while, for ST, the most significant factor is the effectiveness (Figure 3). If the effectiveness of preservative non-free formulations increases, the ICER becomes positive, indicating that preservative non-free formulations would become cost-effective.

## Discussion

In our study, the therapy of POAG+DED consisted primarily of combination therapy, which increased costs for the patients due to co-payment, and partial increases could be observed for the health insurance fund as well. The monotherapy relies mainly on prostaglandins and beta-blockers, while the combination therapy includes all available options. Carbonic anhydrase inhibitors and alfa2 agonists can also be applied as monotherapy though it seems they are rarely prescribed, and in our study, none of the patients were treated with them. According to the European Glaucoma Society Guidelines (EGS), the therapy should begin with monotherapy (27). The aim should be to reach target intraocular pressure (IOP), which is specific for each patient case, depending on the comorbidities of each patient. The level of presence of comorbidities as assessed by Lin et al. demonstrated that no two patients are alike (28), which further underscores the need for individualization of therapy and why the often-neglected co-morbidity of dry-eye syndrome should factor in when physicians are making decisions. In the aforementioned study, the most prevalent accompanying condition was hypertension. According to the EGS Guidelines, the highest reduction of IOP is obtained with prostaglandins, followed by non-selective beta-blockers, alfa adrenergic agonists, selective beta-blockers, and, lastly, topical carbonic anhydrase inhibitors. This could explain the high rate of prescription of PG observed in our study, meaning that Bulgarian physicians respect the guideline recommendations. However, it seems physicians do not exhaust all monotherapy options but would rather move on quickly to combination therapy. If the outcome is not sufficient or the therapy is not well-tolerated, then the option of choice is to switch to another monotherapy rather than adding a second drug. If the initial treatment is well-tolerated but the reduction of IOP is not sufficient to reach the target IOP, then a second drug should be added and so on until a maximal combination is applied (one drug from each of the four therapeutic groups). The fact that, in our study, the combination therapy was prescribed to only a third of patients should indicate that most patients had achieved their target IOP and disease control with monotherapy but had nonetheless developed dry-eye disease.

In view of the high prevalence of comorbidities at older ages and the different responses to treatment by different patients, the authors believe that the need for dry-eye prevention is indisputable. Thus, the main concern facing patients and national health systems should be the cost-effectiveness of treatment strategies. Medical treatment strategies for POAG have long been the mainstay and most preferred modalities because they are comparatively less invasive. There have been many cost comparisons between different therapeutic groups of medicines as first-line medication or monotherapy [e.g., beta-blockers vs. prostaglandin analogs (PGA)], as well as between monotherapy and dual therapy or mixed-combination formulae (29–36). These studies usually measure the cost per mmHg reduction in IOP, but they are not based on real-life data. The emergence of new drugs and the chronic nature of glaucoma have caused a substantial rise in therapeutic cost; however, we did not observe significant differences between the median costs of preservative-containing and preservative-free formulations, as has been observed in other studies as well (37, 38). Although we acknowledge the importance of IOP as a diagnostic criterion and target of treatment, we also believe that the prevention of additional complications such as dry eye should factor in when recommending treatment. Since dry-eye treatment in Bulgaria is not currently reimbursed, the burden experienced by patients is twice heavier than that on the National Health Insurance Fund. The authors argue that dry-eye syndrome should be considered a complication of glaucoma, particularly related to the choice of treatment, which would make it important for physicians to err on the side of caution when choosing medication, especially for elderly patients. Preventing the development of dry eye would have a positive cost-saving effect in future as the disease progresses, since the costs of treatment increases with severity—a trend confirmed for the wider European region by Traverso et al. (39). Despite the costs for Bulgaria being lower than those mentioned in that study and despite the fact that current therapy practices are cost-effective, physicians should also consider lessening the burden on patients.

The burden of non-communicable diseases has a heavy toll on public resources. The share of health care expenditures as a percentage of GDP has been increasing steadily; however, previous studies have observed that there is a large discrepancy in purchasing power between CEE Countries and western Europe, which has manifested in different allocation priorities for resources (40). While many western countries also focus on prevention and diagnosis when financing the health system, the budgetary restrictions in Balkan countries have forced local governments to focus more on medicine costs and cost containment measures rather than incentivizing health promotion, prevention, and primary care (41), despite this leading to episodic rather than continuous care (42). The situation with glaucoma treatment in Bulgaria shows similar tendencies. The fact that preservative-free medication is considered only after patients develop dry eye neglects the possibility that their early adoption in glaucoma treatment can lessen the future burden and reduce costs. Cost studies such as ours provide insight into all of the factors that can impact a disease, are important resources in the struggle of eastern European countries to improve healthcare decisions when prioritizing interventions, and further emphasize the importance of pharmacoeconomic studies for the region.

Our study is the first to explore and model the cost-effectiveness of POAG based on real-life measures of therapeutic effect. Other studies have focused on computer-based simulations based on probabilities from epidemiologic studies (43, 44). To the best of our knowledge, it is also the only study to explore the cost-effectiveness of POAG+DED pharmacotherapy with preservative-free and non-free formulations in Bulgaria, giving important context for the national situation. A similar study exploring the association of dry eye with glaucoma and age found that dry eye usually appears at the age of 50 and is related also to dry mouth, skin, and type of glaucoma. It is observed most frequently in patients with pseudoexfoliation glaucoma, followed by those with POAG (7). The complexity of developing dry-eye disease has been elucidated in previous studies (45), where it was evident that prevalence varies tremendously, as it depends on a multitude of factors. The study by Zhang et al. (45) proposed the concept of the “ocular surface microenvironment,” with multiple systems working together to produce stable tear-film, which prevents the development of the disease. Proper regulation of the homeostasis is key to preventing complications, including vision loss, which again emphasizes the importance of taking measures to prevent complications by prescribing preservative-free formulations. We acknowledge that it is important that physicians have all treatment modalities available to them, and, while the Bulgarian market has access to both preservative-free and non-free therapies, the choice should be made not only with the target IOP in mind but based on multiple other factors, such as age, duration of disease, risk profile, and the financial burden experienced by the patient. Our observations showed that the disease is of significance to elderly people, who are also more likely to have financial difficulties that affect their access to medicine, especially in underfunded regions such as Central and Eastern Europe (46, 47). Thus, the finding that glaucoma treatment comes with a high degree of co-payment is a significant one when added to the finding that there is no significant difference in the cost of the two treatment modalities.

The decision tree showed different values for the incremental cost-effectiveness ratio depending on the type of test used to measure the effect. The best results were seen with preservative-free formulations when the degree of disease control was measured through changes in NIBUT and TMS but not with ST. NIBUT and TMS are related to the lipid phase of the tear film, while ST is associated with the aqueous phase. Therefore, it could be the case that preservatives have a more pronounced effect on the lipids and the meibomian glands rather than on water production—a result supported by investigations into the toxicity of benzalkonium chloride (BAK), which can also increase IOP (48).

Similar to our study is that of Guedes et al., which used a decision analysis model for a 5-year time horizon from the point of view of the Brazilian Public National Health System (49). The study evaluated treatments post application of topical medication and found that non-penetrating deep sclerectomy (NPDS) is less costly and more effective than medical therapy when three topical medications are required (50). The fact that the cost increases with advanced glaucoma, this suggests that therapy is necessary in the earlier stage of glaucoma (47). Taking into account all the factors that influence the ocular surface and progression of disease, we consider that all medications should be evaluated for their potential contribution to the processes that lead to loss of tear film. This is why we believe the oft-overlooked aspect of preservative toxicity requires more attention.

Sensitivity analysis shows that within ±30% variation of the results and costs, preservative-free formulations remain cost-effective. The cost influences the results to a greater extent than the effectiveness of treatment. Therefore, we can conclude that reducing the burden experienced by patients will have a cost-saving effect. For the three selected measures of the result, the ICER is far below three times GDP per capita, which means that preservative-free formulations are a cost-effective therapy.

Many other factors can influence the differences in the cost-effectiveness of glaucoma therapy observed in different countries. This disparity may be due to differences in health care systems (51–53), to life expectancy differences, or to racial differences (54, 55). Although the main focus of our study was the effectiveness of preservative-free formulations from the perspective of the National Health Insurance Fund, the finding that dry-eye disease develops in elderly patients is of interest, alongside the finding that the copayment burden falls mainly on the patients, rather than being paid by institutions.

GDP is an important threshold, according to the WHO. Treatment is moderately effective if the cost is between one to three times GDP per capita. Our results are far below this threshold, as current estimates put the GDP per capita in Bulgaria at 15,226 BGN (€7808), so we can consider the treatment as highly cost-effective for the national healthcare system.

## Limitations

Although this study is the first of its kind to address the issue of dry-eye disease with concomitant primary open-angle glaucoma (POAG), it has several limitations. Firstly, the number of patients, although collected from the largest clinic in the country, is low. More research and wider collaboration will be needed in the future to expand the sample size. Furthermore, patients with previously diagnosed and treated POAG were included, introducing bias since prior therapy was not recorded. We did not include the patient burden of disease (e.g., Quality of Life, days absent from work) or data on financial security when making the analysis. More light may be shed on the effectiveness of treatment when different socio-demographic aspects are addressed, as is patient quality of life.

Additionally, decision tree models are not typically used for chronic diseases, but due to a lack of previous data and no knowledge on other transition probabilities, it was the preferred method for analyzing the costs and outcomes.

Finally, the probability of developing dry-eye disease on preservative-containing medicines has not been investigated. The main focus was on the level of disease control with both classes of medication. The authors plan to address this limitation in further studies, expanding the model to include these calculations, since this could influence the cost-effectiveness of therapy. However, the finding for patients who had already developed dry-eye disease should not be discounted.

## Conclusions

The therapy of POAG+DE with preservative-free formulations is cost-effective when the WHO threshold of three times GDP is considered. Median costs with both treatment modalities, preservative-free and non-free, were similar; however, patients with established dry-eye disease experience a heavy burden in terms of co-payment, which is higher than the burden experienced by the National Health Insurance Fund. Preventing the development of DE could have cost-saving implications. Despite this, both alternatives can be considered cost-effective for the National Health Insurance Fund. Physicians should take into account the copayment aspect and discuss treatment strategies with patients, since our findings suggest that dry-eye syndrome mostly affects elderly glaucoma patients.

## Data Availability Statement

All datasets generated for this study are included in the article/supplementary material.

## Ethics Statement

The studies involving human participants were reviewed and approved by ethical committee at the University Hospital Alexandrovska at the Medical University of Sofia. The patients/participants provided their written informed consent to participate in this study.

## Author Contributions

AV and SK were responsible for patient recruitment, data collection and pharmacotherapy decision. Statistical work and write-up were done by KT.

### Conflict of Interest

The authors declare that the research was conducted in the absence of any commercial or financial relationships that could be construed as a potential conflict of interest.

## Abbreviations

POAG: Primary Open-Angle Glaucoma
DED: Dry Eye Disease
OSD: Ocular Surface Disease
NCPR: National Council on Pricing and Reimbursement
TMS: Total Meiboscore
NIBUT Ave: Non-Invasive Tear Break-Up Time (average)
ST: Schirmer Test
PG: Prostaglandin Analogs
BB: Beta-Blockers
Ca AA: Carbonic Anhydrase Inhibitors
Alfa 2 AA: Alpha-2 Adrenergic Agonists
RI: Redness Index
MGL: Meibomian Gland Loss (%)
IOP: Intra-Ocular Pressure.
